# Highly extended filaments in aqueous gold nano-particle colloidals

**DOI:** 10.1038/s41598-018-24479-9

**Published:** 2018-04-13

**Authors:** Shuai Yuan, Feng J. Liu, Li R. Wang, Jun Y. Nan, Min Li, Bo Q. He, He P. Zeng

**Affiliations:** 1 Shanghai Key Laboratory of Modern Optical System, Engineering Research Center of Optical Instrument and System (Ministry of Education), School of Optical-Electrical and Computer Engineering, University of Shanghai for Science and Technology, Shanghai, 200093 China; 2 China State Key Laboratory of Precision Spectroscopy, East China Normal University, Shanghai, 200062 China

## Abstract

A new regime of filamentation has been discovered in aqueous gold nanoparticle colloidals (AGNC). Different from filamentation in liquids, in this regime, by doping water with gold nanoparticles, there is no observable multiple small-scale filaments, but instead a spatially continuous plasma channel is formed. The length of the filament is more than ten times as compared with that in water. Filamentation in AGNC is characterized by a colorful light channel, with generated supercontinuum ranging from 400 nm to 650 nm which is scattered along a cyan-orange path.

## Introduction

Femtosecond laser filamentation has been observed in all kinds of optical medium, gases, liquids and solids^1–12^. The filamentation in liquids is mainly due to the balance between Kerr self-focusing and defocusing of the plasma generated at high intensities in the self-focal region. The plasma forms as a consequence of multiphoton ionization or multiphoton excitation of electrons from the valence bands to the conduction bands^11–20^. In liquids, a high nonlinear refractive index usually leads to multiple filamentation, because the critical powers in condensed materials are 2–3 orders of magnitudes lower than those in gases. Each hot zone across the beam pattern self-focuses into a child filament if the intensity and power are high enough^1,2^. Many interesting phenomena have been observed such as intensity clamping^21^, shock wave formation^11–13^, cavitation^11^, bubbles generation^13^, and so on. It provides unique capabilities for applications like supercontinuum generation^22^, liquid stirring^23^, nanoparticles production^24^, and microfluidic chips fabrication^25^. The above mentioned applications usually prefer stabilized filaments (multiple filaments) with a long and spatially continuous plasma channel, which actually increases the length of interaction. In order to generate stabilized filaments in liquids, it usually requires tight focusing and high input power (for water, ƒ < 10 cm, P > 40 P_cr_^11^). Without the focal criterion, each child filament evolves separately, and leads to random filament distribution at the cross section of the laser propagation. Besides, although tight focusing of laser beam with a high input power gives rise to several-millimeters-plasma channel with close to critical electron density. In this case, a lot of energy is absorbed by electron collisions and the beam diverges fast after the filament. Thus, methods for generating filaments with longer, stabilized and spatially continuous plasma channel are still in need.

Nowadays, (ns/fs) laser ablation benefits noble metal nanoparticles generation with small size, and high purity, especially gold nanoparticle (colloidal gold)^24^. Colloidal gold has shown strong local field enhancement and large optical nonlinearity under light illumination^26,27^, which has extensive applications in various fields e.g. toxic gas detection^28^, tumor detection^29^, drug delivery^30^, and gene therapy^31^. Laser filamentation in AGNC is believed to involve mechanisms or procedures as follows^11,23,27,32,33^. (i) The fundamental wave resonates with the plasmon of gold nanoparticles, which enhances nonlinearity of the media, (ii) the temperature increases locally in water, which leads to shock wave generation and water convection (laser-induced stirring), (iii) the gold nanoparticles are promising catalysts, which might benefit chemical reactions under illumination of femtosecond laser pulse in the future. In this sense, it provides a perfect scheme to investigate the filamentation in AGNC.

By creating laser filamentation in water doped with gold nanoparticles (AGNC, nanospheres with d = 12 nm), we generate longer filament. The filamentation in AGNC can be initiated with a looser focusing and lower power than that in water. The slow variation of the white light along the filament indicates a spatially continuous and stable plasma channel. The longitudinal white light distribution for filamentation in AGNC is also investigated.

## Experimental Setup

**Figure 1:**
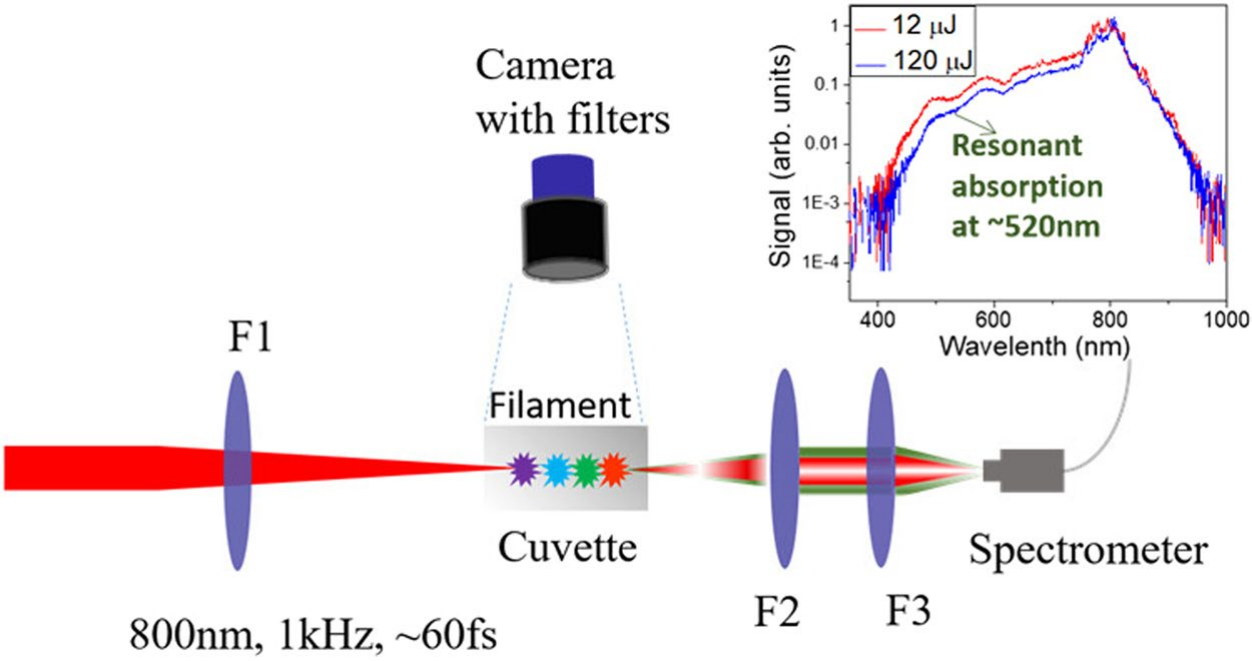
Schematic of the experimental setup. The laser pulses were focused by a fused silica lens F1 (ƒ = 20 cm) into AGNC/water. Filament inside the solution was imaged by a CCD camera with ND & bandpass filters in front. The output emission was collected by two fused silica lenses F2 (ƒ = 20 cm) and F3 (ƒ = 8 cm) to a fiber coupled spectrometer. The inset shows spectra obtained for the propagation of the laser pulse in AGNC with 12 and 120 μJ for input pulse energies.

The experimental setup is shown in Fig. 1. The femtosecond laser system, which consisted of a Ti:sapphire oscillator and a regenerative amplifier, delivered 800 nm (λ_0_), 60 fs, linearly polarized pulses with a maximum pulse energy of 2.4 mJ at 1 kHz repetition rate. The power of the laser pulse was adjusted by a variable attenuator. The filament was created in AGNC in the middle of a fused silica cuvette by focusing the output laser pulse with a lens (F1, ƒ = 20 cm). A CCD camera (1280 × 1024 pixels) was placed on the top of the cuvette in order to project the filament in an area of about ~100 mm × 80 mm. The CCD camera was gated with an exposure time of 100 milliseconds. After the filament, the generated supercontinuum emission was collimated by two fused silica lenses and collected by a fiber-coupled spectrometer (Ocean Optics HR4000CG). Appropriate neutral density (ND) filters were put in front of the spectrometer in order to avoid CCD saturation. The generated supercontinuum in AGNC exhibits a dip at around 520 nm as shown by the inset in Fig. 1, which corresponds to the resonant wavelength of the gold nanospheres with d = 12 nm.

## Results

**Figure 2:**
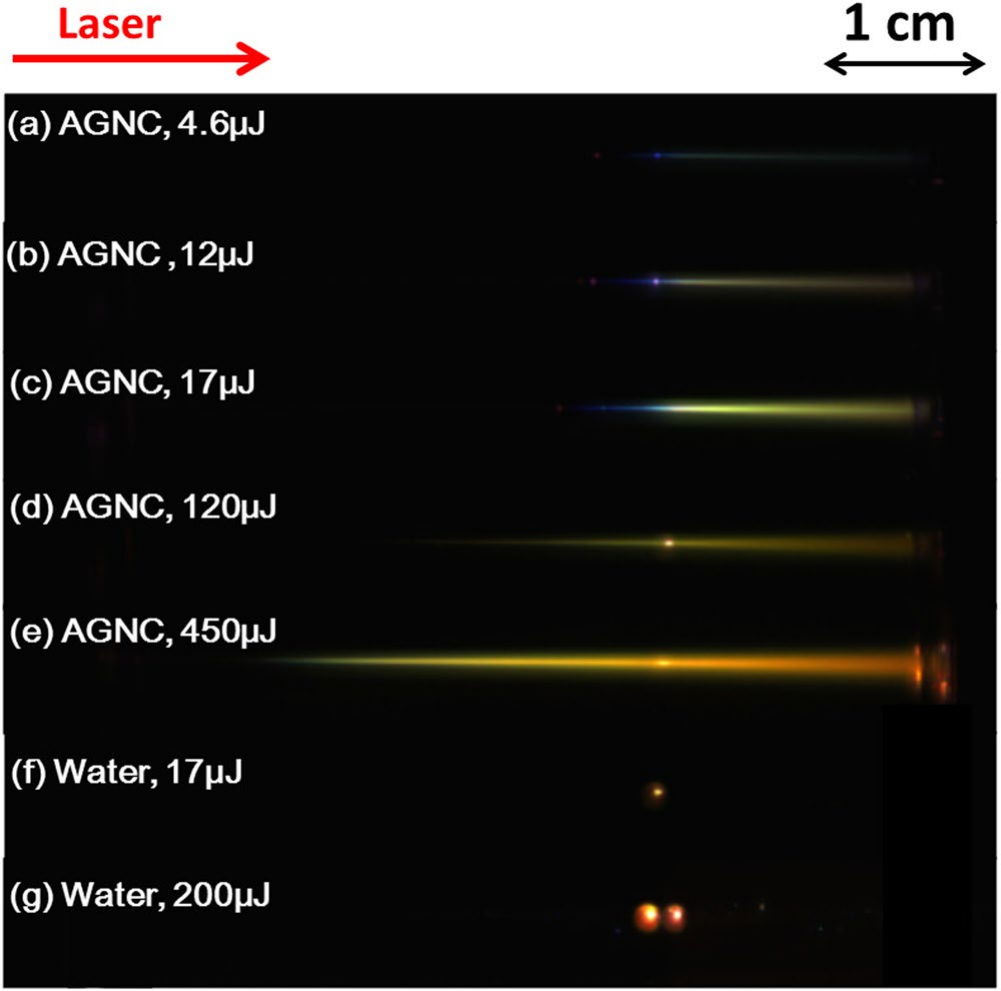
Top views of light channel in AGNC with pulse energies of (**a**) 4.6, (**b**) 12, (**c**) 17, (**d**) 120, (**e**) 450 μJ, and in water with pulse energies of (**f**) 17, (g) 200 μJ. During the experiment, in (**a**–**c**) no filters were placed in front of the CCD camera, while in (**d**–**g**) we use one ND filter to avoid the saturation of the images (30% transmission). The input pulse energies were measured right after the focusing lens (F1 in Fig. 1).

Figure 2(a–e) show the top views of filaments generated in 12-nm AGNC with input pulse energies of 4.6~450 μJ. The generated white light is scattered along the filament. It is important to note that powers corresponding to all these energies are beyond 10 times of critical power (P_cr_ = 4.2 MW). It means that the plots in Fig. 2 represent multiple filaments. In Fig. 2(a–e), with an increase of the input pulse energies, the onsets of filaments start at earlier positions. Filamentation in AGNC is characterized by a spatially continuous plasma channel, with the length from millimeters to centimeters. The longitudinal color distribution of the filament from the beginning to the end gradually changes from cyan to green and then to orange. “Bright dots” can be observed as well for filamentation in both AGNC and water, which might indicate regions of high intensity, owing to the nonlinear Kerr foci and re-foci^17^. Different from a spatially continuous plasma channel for filamentation in AGNC, filamentation in water is featured by discrete plasma channels with the length from hundreds of microns to millimeters [see Fig. 2(f,g)].

**Figure 3:**
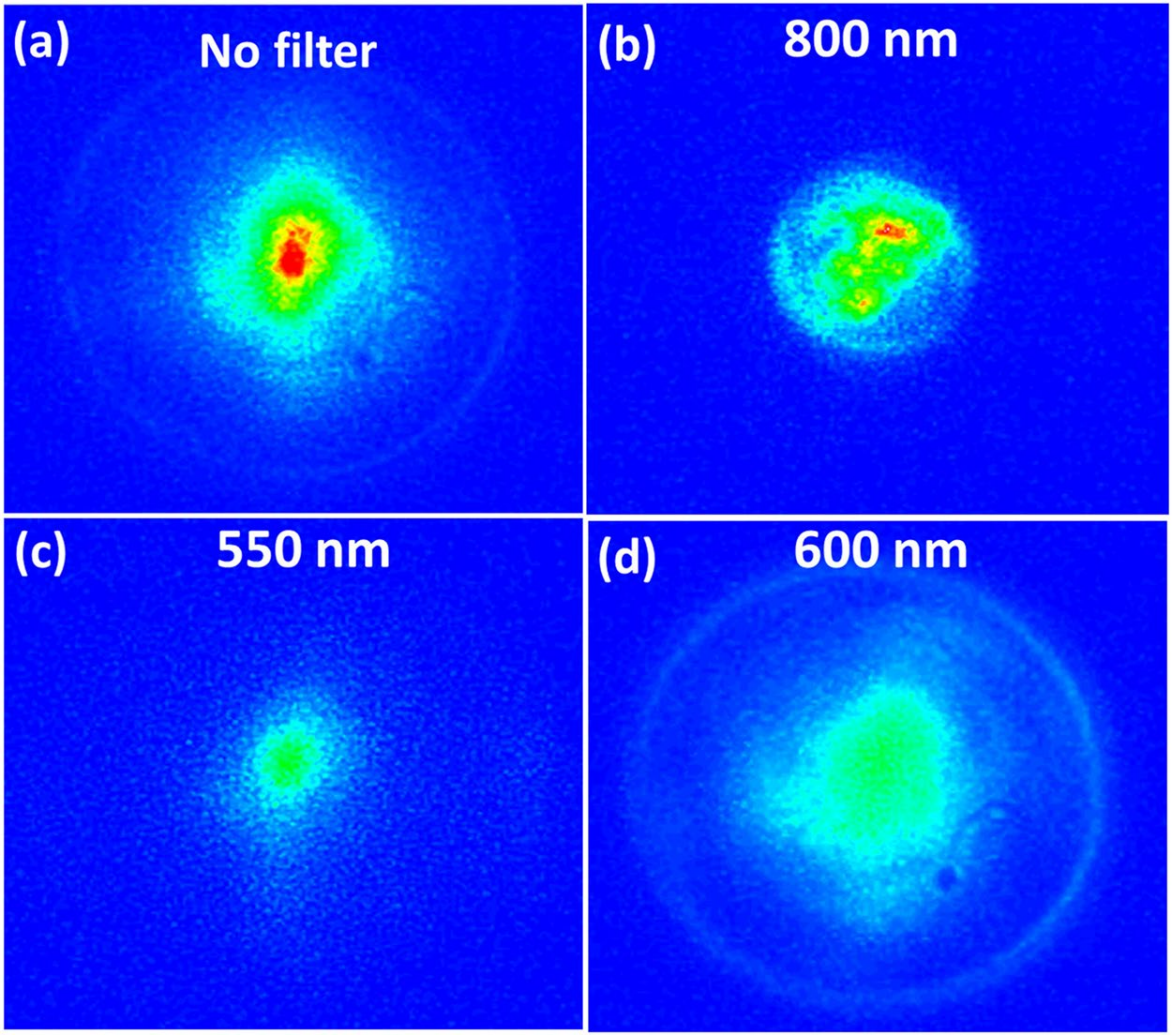
Beam patterns measured after the filament in AGNC with input pulse energy of 6 μJ using a CCD camera with no bandpass filters (**a**), bandpass filter centered at 800 nm (**b**), 550 nm (**c**) and 600 nm (**d**). Here the bandpass filters have 40-nm transmission bandwidth.

The generated white light (supercontinuum) at the end of the filament in AGNC covers a broad spectral range, from 400 nm to 650 nm. The beam patterns measured after the filament are shown in Fig. 3. In Fig. 3(a), the hot spot in the center at 780–850 nm reconfirms self-guided filamentation^34–37^. In Fig. 3(b), beam pattern of fundamental frequency components at λ_0_ = 800 nm splits into several parts, which indicates multiple filamentation. While the generated white light has larger divergence with the strongest component around 600 nm [see Fig. 3(d)].

**Figure 4:**
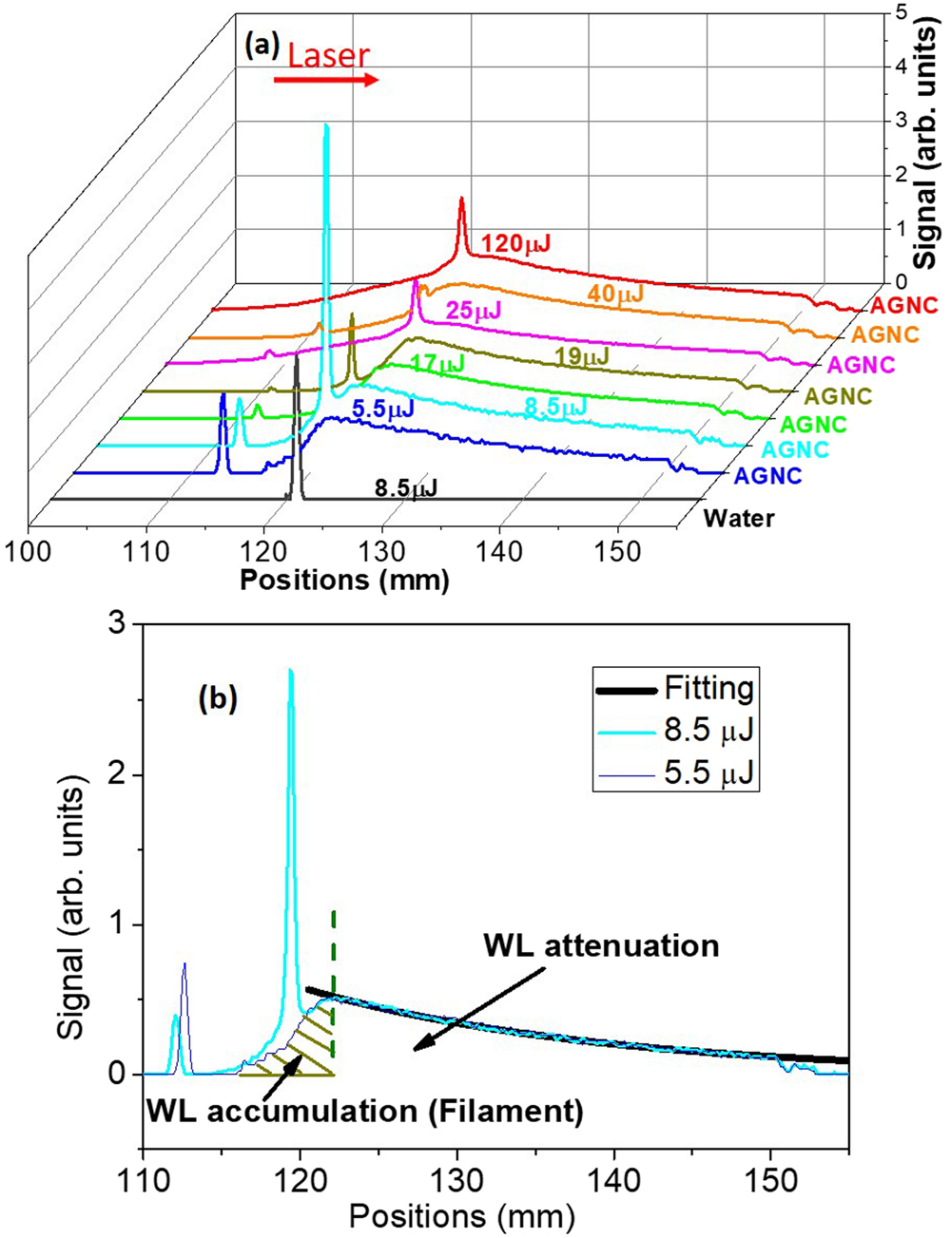
(**a**) The generated white light distributions along the filament in water and AGNC under different pulse energies. Each curve in AGNC is normalized to the peak of the “envelopes”. (**b**) White light distributions in (**a**) with input pulse energies of 5.5 and 8.5 μJ are shown as an example. In (**b**), two “envelopes” have similar trailing edges. Thus, we use one decay curve (the black solid curve) to exponentially fit the two. During our experiment, we put a bandpass filter (340–680 nm for the transmission band) in front of the CCD camera, in order to eliminate the fundamental-wave components at λ_0_ = 800 nm. WL: white light.

The white light distributions along the filament are investigated in Fig. 4 in order to compare the evolution of filaments in AGNC and those in water. The distributions were measured by taking the top view of light path by the CCD camera. Note that the intensity of white light does not equal to the intensity of laser field. In Fig. 4(a), under different pulse energies, the white light along light path in AGNC consists of a broad envelope and several identical spikes. For the sake of simplification, they are named as “envelope” and “spikes” in what follows. The “envelope” of the white light in AGNC is characterized by a rising edge with “spikes” and a slow trailing edge. The trailing edge exhibits an exponential decay. While in water, only identical spikes could be observed.

The generated white light ranging from 400 nm to 650 nm is due to Self-phase modulation and the plasma generation^21^. On one hand, through propagation, within the scale of the filament, the generated white light was accumulated over the interaction length (approximate filament length). It gave rise to the enhancement of the strength of white light along the filament. On the other hand, the generated white light was attenuated, when it propagated through AGNC. Several reasons could be attributed to an exponential decay of the white light distribution along the propagation axis, such as linear absorption due to a resonance with H_2_O molecule vibrations (about 0.9 cm^−1^), Rayleigh scattering induced by 12-nm nanoparticles, stimulated Brillouin/Raman scattering, resonant absorption, and so on.

### Longitudinal White Light Distribution for Filamentation in AGNC

When the input peak power (P) is far above the critical power (P_cr_) for self-focusing, the modulational instability breaks up the beam into a large number (${\rm{N}}\approx {\rm{P}}/{{\rm{P}}}_{{\rm{c}}{\rm{r}}}$) of filaments^2,38^. In our experiment, the peak power that we used during the experiment related with Fig. 4 is more than ten times of the critical power in water (14P_cr_ ~ 312P_cr_), which also fits the criterion of mutifilamentation. In AGNC, the white light was scattered mainly by 12-nm nanoparticles in every unit length along the propagation axis. Thus, it could be observed by naked eyes from the side. Let’s consider Fig. 4(b) from the left to the right along the propagation axis. Firstly, within the scale of the filament, in each unit length, the rate of white light generation is likely to be larger than its attenuation (absorption/scattering). Thus, more white light is accumulated as propagation distance increases, which is shown by the rising edge of the “envelope”. It is indicated as WL accumulation in Fig. 4(b) (shadow region). After that, roughly corresponding to the end of the filament, when the beam starts to diverge, the intensity decreases. As a result, less white light was generated in each unit length along the axis. At some point, when the attenuation (absorption/scattering) rate of white light balances its generation (accumulation) rate, it is correlated with the maximum of the white light “envelope” in Fig. 4(b). Then after the filament, the intensity decreases. The attenuation rate of white light is larger than its generation rate. Hence, after the filament, the attenuation dominates the on-axis distributions of white light. In this sense, for the trailing edge of the “envelopes” of the Fig. 4, the intensity of white light versus on-axis distances exhibits an exponential decay. Therefore, the filament maintains its high intense part at the region of WL accumulation in Fig. 4(b).

Although the trailing edges of the “envelopes” in Fig. 4(a) start at different positions, they can be exponentially fitted with similar attenuation coefficients of 0.039 ± 0.005 mm^−1^ [see Fig. 4(b)]. In AGNC, the white light was scattered by 12-nm nanoparticles, while in case of filament in distilled water, the white light could not be strongly scattered after the filament. Thus, in case of filamentation in water, only spikes could be observed. In Fig. 4, the length of high intense part of the filaments in AGNC is roughly estimated by the longitudinal scales covered by the rising edge of white light distribution [see the shadow region in Fig. 4(b)]. Although the filament might still undergo beyond the shadow region in Fig. 4(b), the shadow region indicates its strongest part. The length of the filaments (WL accumulation region) in Fig. 4 was estimated to be 5.9, 6.2 and 18.8 mm for the input pulse energies of 5.5, 8.5, and 120 μJ, respectively. On the opposite case, filamentation in water is unstable and discrete. In our experiment, the length of filaments in water was measured to be hundreds of microns to 1.8 mm, as we increased the energy from 1.5 to 200 μJ, by taking the range of the longitudinal distribution of white light in Fig. 4(a). It was roughly an order of magnitude shorter than that in AGNC.

### Colorful light path for filamentation in AGNC

**Figure 5:**
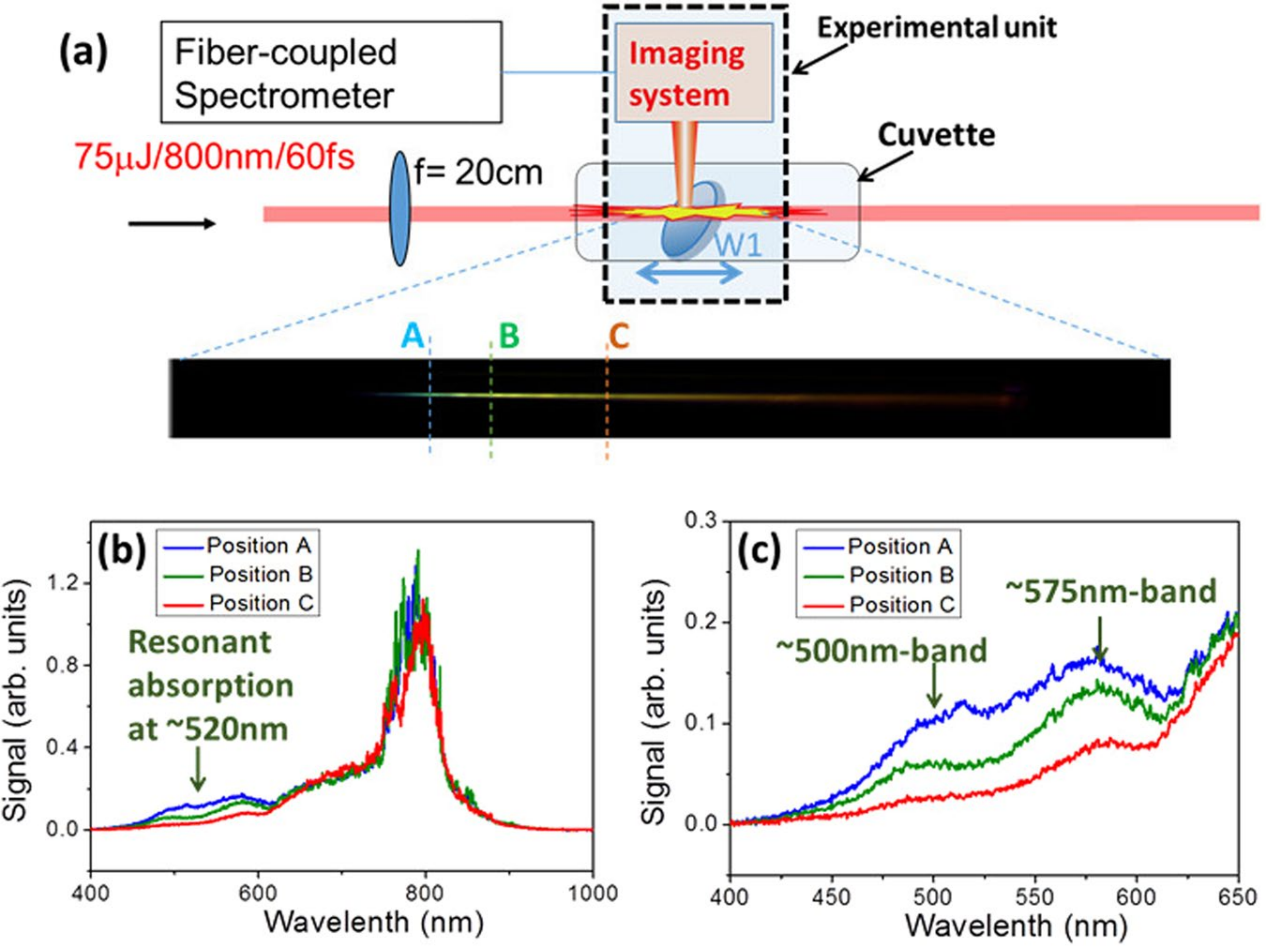
(**a**) A fused silica wedge (W1) was moved along the filament in AGNC, which stopped the filament at different positions (Positions A, B and C). (**b**) The on-axis spectra at each position. The spectra at different positions are normalized to the intensity of the signal at 800 nm. Here, a longer cuvette with 150 mm × 20 mm × 20 mm in size was used. (**c**) The spectra in (**b**) with the spectra ranging from 400 nm to 650 nm magnified.

For filamentation in AGNC, the longitudinal color distribution of the filament from the beginning to the end gradually changes from blue to green and then to orange. One supplementary experiment was carried out by measuring the generated supercontinuum inside the filament, in order to investigate the role of scattering in AGNC. An experimental unit was moved along the beam. It stopped the filament by a fused silica wedge at different positions [see Fig. 5(a)]. For each position, the weak reflection from the wedge was directed upward out of the cuvette. We call these on-axis spectra. After that, the on-axis spectra were collimated by fused silica lenses and directed to the spectrometer with several ND filters in front. The spectra at Positions A, B and C are shown in Fig. 5(b). In Fig. 5(b), supercontinuum covers a broad spectral range from 400 nm to 650 nm.

In AGNC, 0.004 mm^−1^ is the linear attenuation coefficient. While the trailing edges of the “envelopes” in Fig. 4(a) are exponentially fitted with attenuation coefficients of 0.039 ± 0.005 mm^−1^. It indicates that other processes like resonant absorption have also contributed the energy losses inside the filament. The white light was scattered mainly by ~12-nm nanoparticles through Raleigh scattering, which could be observed perpendicular to the propagation axis with naked eyes.

The colorful light path is due to the scattering of the supercontinuum inside and after the filament. The strength of Rayleigh scattering is proportional to ~*λ*^−4^. When the filament was created, the generated plasma induced blue shift of the spectrum. It was indicated in Fig. 5(c) by two strong emission bands around 500 nm and 575 nm. Due to Raleigh scattering, higher frequency components were scattered. Thus, at the beginning of the filament at Position A in Fig. 5(a), more ~500-nm band was scattered and could be observed. Accordingly, at Position A the color of the light path was cyan. After that, on one hand the generated supercontinuum still propagated and had experienced more attenuation for higher frequency components due to Rayleigh scattering, before it arrived at Position B in Fig. 5(a). On the other hand, the energy losses of the fundamental-wave beam at λ_0_ = 800 nm led to less blue frequency shift as the beam propagated forward. Thus, when the laser pulse arrived at Position B, the emission band at ~500 nm had attenuated more than the band at ~575 nm, which was shown as the green solid curve in Fig. 5(c). In this case, more ~575-nm band was left and scattered at Position B. Hence, the color of the light path changed to green-yellow at Position B. As the same reason, the higher frequency components decreased more when the pulse still propagated forward, which gives rise to the orange path at Position C in Fig. 5(a). Generally speaking, along the propagation axis higher frequency components are easily scattered. Meanwhile, energy dissipation also decreases the intensity along the light path and less high frequency components are generated at forward positions. Thus, color changes spatially along the light path. Therefore, both Raleigh scattering and energy dissipation are essential for the formation of a colorful light path. During the experiment, we also launched the filament in colloidal copper or silver solution with the average particle size of ~50 nm. Then colorful light path was observed as well. We claim that the colorful light path can be a universal phenomenon for filamentation in nanoparticle-doped solutions.

## Discussion and Conclusion

In conclusion, we have experimentally investigated filamentation in water doped with 12-nm gold nanoparticles. In AGNC, a longer and spatially continuous plasma channel is formed. Stronger and spatially continuous filament has been implemented in various tasks. For instance, it increases the interaction distances between the filament and the medium, which will benefit for filament-induced chemical reactions. In addition, we want to point out that many questions remain open in the physics for filamentation in AGNC, e.g. the effect of local field enhancement of gold nanoparticles on the filamentation and how the photothermal effect creates the water vapor and induces the water convection.

## Methods

The cuvette used in our experiment was 100 mm × 20 mm × 20 mm in size, 1.5 mm for the thickness of each side and open on top. The AGNC were purchased from Huzheng company (Shanghai). In AGNC, the diameter of gold nanoparticles (nanospheres) was about 12 nm as measured by using scanning electron microscopy. They were evenly suspended in distilled water with a concentration estimated to be 200 ppm.

During the experiment, we used two cuvettes of the same size. One was filled with distilled water. The other was filled with AGNC. First, the filaments were investigated in distilled water under different input pulse energies. We took the top views and the output spectra of the filaments. Afterwards, the same measurements were repeated in AGNC.
